# Addressing pollution challenges for enterprises under diverse extreme climate conditions: artificial intelligence-driven experience and policy support of top Chinese enterprises

**DOI:** 10.3389/fpubh.2024.1436304

**Published:** 2024-09-05

**Authors:** Jingjing Sun, Xin Guan, Yanzhao Zeng, Jiali Zhang, Xiaodie Chen, Xi Zhan

**Affiliations:** ^1^School of Public Administration, Guangzhou University, Guangzhou, China; ^2^Guangzhou Xinhua University, Dongguan, China; ^3^School of Economics and Statistics, Guangzhou University, Guangzhou, China; ^4^School of Management, Guangzhou University, Guangzhou, China

**Keywords:** top Chinese enterprises, environmental protection, diverse extreme climate, artificial intelligence technology, policy support

## Abstract

**Introduction:**

This study investigates the experiences of leading Chinese companies in environmental conservation under varying extreme climate conditions, focusing on the role of artificial intelligence (AI) and governmental assistance.

**Methods:**

A survey was conducted involving 200 participants to assess recognition and endorsement of AI’s role in environmental protection and to explore the adoption of AI technologies by firms for enhancing environmental management practices.

**Results:**

The survey revealed widespread recognition of Tencent’s green initiatives and strong support for AI’s role in environmental protection. Many firms are considering adopting AI technologies to optimize energy management, deploy intelligent HVAC systems, and improve the operations of data centers and smart lighting systems.

**Discussion:**

The findings highlight a strong belief in AI’s potential to advance environmental protection efforts, with a call for increased governmental support to foster this development. The study underscores the importance of a partnership between businesses and governments to leverage AI for environmental sustainability, contributing significantly to conservation efforts.

## 1 Introduction

In today’s world, climate change has become a global focus, with the frequency and intensity of extreme weather events increasing, posing significant challenges to human society and ecosystems (1). In this context, reducing emissions and pollution has become an urgent task for governments and businesses worldwide. However, businesses face diverse extreme climate conditions, such as high temperatures, droughts, and floods, which bring greater complexity and difficulty to pollution management (2, 3). Against this backdrop, the application of artificial intelligence (AI) technology offers a novel solution for businesses (4). AI can assist companies in more effectively managing emissions and pollution, reducing environmental impact through means such as data analysis, model building, and predictive optimization. Especially in large countries like China, some leading enterprises have demonstrated successful experiences in addressing pollution challenges driven by AI (5, 6).

The increasing frequency and severity of extreme weather events exacerbate pollution issues, posing significant challenges to businesses operating in different geographical locations. For instance, companies located in heatwave-prone areas face issues of increased emissions due to heightened energy consumption for cooling purposes. Similarly, enterprises situated in flood-prone regions must address the risk of pollutants being washed into water bodies, potentially harming ecosystems and human health. Additionally, droughts disrupt industrial processes reliant on water resources, compelling businesses to explore alternative production methods that may inadvertently increase pollution.

Faced with such multifaceted challenges, traditional pollution management strategies often prove insufficient, necessitating the utilization of technological advancements such as AI to adopt innovative approaches. By employing AI-driven solutions, enterprises can gain real-time insights into environmental conditions and pollution levels, enabling them to initiate preventive measures. For example, AI algorithms can analyze meteorological data, forecast weather patterns, and anticipate potential pollution hotspots, allowing businesses to take proactive measures to minimize environmental impacts. Against this backdrop, Huang and Wei (7) explored the impact of Chief Executive Officer (CEO) green experience on corporate environmental and social responsibility, demonstrating that the green leadership of top management, when combined with AI technology and policy support, significantly enhanced a company’s performance in pollution management. This provided theoretical support for this experiment’s analysis of how leading Chinese companies, guided by green leadership, leveraged AI technology to address pollution challenges. Alzoubi and Mishra (8) examined the potential and challenges of green artificial intelligence, providing context for this experiment’s discussion on AI-driven corporate pollution optimization. Their study highlighted the broad application potential of AI in environmental protection while also emphasizing the obstacles and challenges that needed to be overcome in the process of technology adoption.

He et al. (9) further demonstrated how a CEO’s early life experiences can influence a company’s green innovation. This experiment is of great significance for exploring how the background of top management affects corporate pollution response strategies under diverse extreme climate conditions, providing a reference for this experiment’s analysis of how corporate leaders, in complex environments, drive environmental innovation through AI technology. Additionally, Akhtar et al. (10) discussed the personalized applications of AI and generative AI in the circular economy, showcasing the potential and diverse application scenarios of AI technology in achieving corporate pollution optimization. This offers a valuable comparative reference, illustrating that AI technology can not only improve corporate pollution management efficiency but also promote the development of the circular economy.

In summary, although existing research has explored the applications and challenges of AI technology in corporate pollution management from different perspectives, there is still a lack of systematic research on how leading Chinese companies optimize pollution management through AI technology and policy support in response to extreme climate conditions. Therefore, the motivation is to fill this gap by systematically analyzing and validating how AI-driven technology and policy work together to help leading Chinese companies address increasingly severe environmental challenges and achieve sustainable development. Furthermore, AI-driven monitoring systems can continuously assess emissions generated by industrial activities, prompting timely interventions to prevent pollution incidents and ensure regulatory compliance. Therefore, integrating AI technology into pollution management strategies offers a promising pathway for enterprises to effectively address the complexity of diverse extreme weather conditions.

The aim of this study is to explore solutions to pollution challenges for enterprises under diverse extreme climate conditions by studying the pollution experiences of top Chinese enterprises driven by AI and drawing lessons from them to provide references for other enterprises and governments. The main contributions and innovations of this study are as follows:

1. Focus on extreme climate response strategies of leading Chinese companies: This study is the first to systematically analyze how leading Chinese companies optimize pollution management through AI technology and policy support under various extreme climate conditions. The innovation lies in combining the study of extreme climate conditions with corporate pollution management, offering a new perspective for related research in this field.
2. Integration of multi-dimensional research methods: This study combines case studies, policy analysis, and empirical surveys, employing a multi-dimensional research approach to validate the effectiveness of AI technology in corporate pollution management. This innovative methodology provides richer and more comprehensive research findings.
3. Emphasis on practical application and policy guidance: Beyond theoretical exploration, this study underscores the importance of practical application and policy guidance. It offers specific recommendations on how companies can better utilize AI technology for pollution management under extreme climate conditions, while also providing a reference for governments on how to formulate and implement relevant policies.

## 2 Methods

Before discussing how companies can effectively manage pollution under diverse extreme climate conditions, this section systematically introduces the methods used. This includes analyzing the challenges of pollution management, understanding the specific impacts of different extreme climate conditions on pollution management, and proposing corresponding solutions. These methods can provide a solid foundation for the study and lay the foundation for subsequent empirical analysis.

### 2.1 Challenges of pollution management under diverse extreme climate conditions

In the face of diverse extreme climate conditions, enterprises encounter a series of complex challenges in pollution management. These challenges stem from the direct impact of climatic conditions on the pollution discharge process; for instance, extreme temperature fluctuations may lead to equipment failures or increased energy consumption. This section explores the nature of these challenges in detail and their specific effects on corporate pollution management strategies, thereby providing theoretical support for understanding the management difficulties posed by extreme climate conditions.

#### 2.1.1 Impact of different extreme climate conditions on pollution management

The impact of different extreme climate conditions on pollution management is a complex and significant issue. Under extreme climate conditions, enterprises face a series of challenges and difficulties in pollution management, stemming not only from the climate itself but also from its impact on infrastructure, resources, and personnel (11). This study analyzes the impacts from three aspects: high temperatures, droughts, and floods.

Firstly, the impact of high temperatures on pollution management is manifested in several aspects. High temperatures can lead to equipment overheating and failure in industrial production, increasing the difficulty of emission control (12). Additionally, high temperatures may result in water scarcity, affecting wastewater treatment and recycling, thereby increasing the difficulty and cost of wastewater discharge. Furthermore, high temperatures can lead to the diffusion and increased concentration of air pollutants, posing challenges in air quality management and threatening human health and the environment (13).

Secondly, the impact of drought on pollution management primarily lies in water scarcity and deteriorating water quality. During drought conditions, the availability of water for production and domestic use is limited, severely affecting wastewater treatment and discharge (14). Moreover, drought may lead to deteriorating water quality, increasing the difficulty and cost of wastewater treatment. Hence, under drought conditions, enterprises need to adopt stricter water-saving measures, enhance wastewater treatment and recycling, and reduce dependence on water resources (15).

Thirdly, the impact of floods on pollution management includes damage to production facilities and infrastructure, as well as the influence on wastewater discharge. Floods can damage enterprise production facilities, causing production interruptions and increasing the difficulty and cost of pollution management (16). Additionally, floods may submerge or damage wastewater treatment facilities, thereby affecting the effectiveness of wastewater treatment and the quality of discharge. Consequently, under flood conditions, enterprises need to strengthen flood prevention measures for facilities and equipment to ensure the normal operation of pollution control facilities and minimize environmental impact (17).

These challenges extend beyond direct physical and environmental impacts to include effects on business operations, supply chain management, and long-term sustainability strategies. Firstly, companies need to recognize the unpredictability and variability of extreme weather events, requiring them to have the ability to adapt flexibly to environmental changes. For example, by establishing more flexible supply chains to address interruptions in raw material supply caused by extreme weather conditions. Additionally, companies can reduce reliance on single resources by diversifying their sources of energy and water supply, thereby mitigating risks associated with extreme weather.

Secondly, strengthening human resource management is also crucial in addressing pollution management challenges under extreme weather conditions. Maintaining employee safety and health in high temperatures or other adverse weather conditions requires companies to develop and implement stricter safety measures and health monitoring policies. Moreover, training employees on environmental protection and awareness of sustainable development is an effective way to enhance the overall capacity of companies to respond to challenges posed by extreme weather.

Lastly, companies should actively explore and adopt innovative technologies and methods to improve pollution management efficiency and effectiveness. In addition to leveraging AI and big data technologies, innovations in sustainable production techniques, clean energy, and resource recycling should also be considered. For example, by adopting more environmentally friendly production processes and materials, companies can not only reduce their direct impact on the environment but also establish a sustainable brand image in the market, thereby gaining a competitive advantage in the long term. Therefore, facing pollution management challenges under extreme weather conditions, companies need to adopt comprehensive strategies covering various aspects such as technological innovation, operational flexibility, human resource management, and long-term sustainable development planning.

#### 2.1.2 Special challenges and difficulties faced by enterprises under different extreme climate conditions

Under diverse extreme climate conditions, enterprises may encounter a series of special challenges and difficulties in pollution management. These challenges involve production, environmental protection, resource utilization, and other aspects, as shown in Table 1.

**Table 1:** Challenges faced by enterprises under three extreme climate conditions and corresponding countermeasures.

Conditions	Challenges	Countermeasures
High temperatures	Decreased equipment stability, prone to malfunctions or overheating; increased air conditioning and refrigeration energy consumption.	Implement effective equipment maintenance and energy management measures.
Drought	Water scarcity affecting wastewater treatment and recycling effectiveness; deterioration of water quality.	Strengthen water resource management, adopt water-saving measures, enhance wastewater treatment and recycling.
Floods	Damage to facilities and equipment, submersion or damage to production and pollution control equipment.	Strengthen flood prevention measures for facilities and equipment, ensure the effective operation of equipment and pollution control facilities.

In Table 1, under high temperature conditions, enterprises face the challenge of decreased equipment stability. High temperatures can lead to equipment malfunctions or overheating, thereby affecting the normal operation of production and pollution treatment. Additionally, high temperatures increase the energy consumption of air conditioning and refrigeration equipment, thus increasing the company’s energy consumption and emissions (18, 19). Therefore, enterprises need to implement effective equipment maintenance and energy management measures to ensure normal equipment operation and reduce emissions. Under drought conditions, enterprises face the challenges of water scarcity and deteriorating water quality. Drought can result in inadequate water supply for production and domestic use, affecting the effectiveness of wastewater treatment and recycling. Moreover, drought may lead to deteriorating water quality, increasing the difficulty and cost of wastewater treatment (20, 21). Hence, enterprises need to strengthen water resource management, adopt water-saving measures, enhance wastewater treatment and recycling to reduce dependence on water resources and emissions. Under flood conditions, enterprises face the challenge of facility and equipment damage. Flooding can lead to the submersion or damage of enterprise production facilities and pollution control equipment, affecting the normal operation of production and pollution treatment. Furthermore, flooding may cause wastewater treatment facilities to fail, increasing the difficulty and cost of wastewater discharge. Therefore, enterprises need to strengthen flood prevention measures for facilities and equipment to ensure the normal operation of equipment and the effective operation of pollution control facilities.

### 2.2 Experience of AI-driven practices in top Chinese enterprises

The importance and leading position of top Chinese enterprises in environmental protection are reflected in several aspects, including technological innovation, management practices, and policy compliance. The proactive practices and achievements of these enterprises in the field of environmental protection play a crucial role in driving the development of the entire industry and improving the level of environmental protection. Top Chinese enterprises refer to companies that perform exceptionally well in the Chinese market, significantly influencing technological innovation, economic scale, and environmental protection. The experiment quantifies and selects these companies based on the following criteria. Financial Indicators: The primary basis includes financial data such as annual revenue, profit, and total assets. This data is sourced from the China Statistical Yearbook and related annual reports. To ensure data accuracy and relevance, the average values from the most recent 3 years (2021–2023) were used to reflect the companies’ stable performance and sustained market influence. In addition to financial data, this study also analyzes the companies’ market share within their industries, technological innovation capabilities, and their investments and achievements in environmental protection. These indicators are obtained through industry reports, company annual reports, and evaluations from third-party assessment agencies. For the evaluation of environmental practices, the study examines the specific measures and outcomes related to environmental protection and the application of green technologies. This information is sourced from the companies’ annual environmental reports, industry awards, and recognitions.

The application of AI technology in addressing pollution challenges is becoming an important approach to solving environmental issues. AI technology, through methods such as data analysis, model building, and intelligent decision-making, can assist enterprises in more effectively managing emissions and pollution, thereby reducing environmental impact (22). The following are the main directions of AI application in pollution challenges:

Artificial intelligence plays an important role in pollution monitoring and prediction. By utilizing technologies such as sensor networks, drones, and satellite data, AI can monitor environmental indicators such as air quality, water quality, and soil in real time, predict the spread of pollutants and their impact, and help enterprises take timely measures to reduce environmental damage (23). AI has broad applications in pollution control and management. By establishing pollutant emission models and environmental impact assessment models, AI can optimize pollution management schemes, improve pollutant treatment efficiency, and reduce environmental risks. Additionally, AI can achieve intelligent management of pollution control facilities through intelligent monitoring and control systems, reducing human intervention and improving operational efficiency (24). AI can also play a role in environmental remediation and resource recycling. By analyzing the pollution status of soil and water bodies, AI can design more effective environmental remediation schemes, improve remediation efficiency. Simultaneously, AI can optimize the process of resource recycling, improve resource utilization efficiency, and reduce environmental impact (25).

Building upon this foundation, AI technology holds tremendous potential in advancing the development and application of green and low-carbon technologies. AI not only reduces a company’s carbon footprint by optimizing energy consumption and improving energy efficiency but also plays a significant role in areas such as new energy development, carbon capture, and storage technologies. Through deep learning and machine learning algorithms, AI can analyze and predict energy demand, optimize energy production and distribution, thus supporting the wider adoption of renewable energy and the achievement of carbon reduction goals. Additionally, AI plays an important role in promoting the formulation of environmental policies and regulations, strengthening public awareness of environmental protection, providing robust technological support, and innovative solutions for achieving sustainable development goals.

### 2.3 Policy support from the Chinese government in environmental protection and AI development

The Chinese government has been actively promoting related policies in environmental protection and AI development, aiming to promote the application and development of environmental protection and AI technology. In the aspect of environmental protection, the Chinese government has enacted a series of environmental protection laws, regulations, and policy documents, strengthening management in areas such as pollutant emissions, environmental monitoring, and environmental impact assessment. For example, China has implemented key environmental protection actions such as the Action Plan for Air Pollution Prevention and Control, Action Plan for Water Pollution Prevention and Control, clearly defining governance goals and measures, thereby promoting the advancement of environmental protection work.

In terms of AI development, the Chinese government also attaches great importance and has introduced a series of supportive policies. China has issued the “Next Generation AI Development Plan,” proposing development goals and roadmaps, and clarifying the application directions and key areas of AI technology in various economic and social fields. Additionally, China has also implemented a series of policies to support the AI industry, including tax incentives, research funding support, talent cultivation, and other support measures, promoting innovation and application of AI technology. To promote the integration of environmental protection and AI technology, the Chinese government has also introduced a series of supportive policies. For example, encouraging enterprises to use AI technology for environmental monitoring, pollution control, and improving environmental protection efficiency and levels. Meanwhile, the government supports research institutions and enterprises to conduct applied research on AI in the environmental field, promoting innovation and development of AI technology in the field of environmental protection.

### 2.4 Case analysis

This study conducts a case analysis using “Tencent’s Environmental Practices Driven by AI” as an example. Tencent, as a leading technology company in China, has also demonstrated active practice and innovation in the field of environmental protection. Tencent continuously optimizes the energy efficiency management of its data centers through the application of AI technology, achieving the goal of energy conservation and emission reduction. The experiment selected Tencent because, as a leading technology company in China, Tencent’s innovations and applications in environmental practices are highly representative. By using Tencent as a case study, the study aims to provide valuable insights and references for other companies. Tencent has widely applied AI technology in various areas, including energy efficiency management, smart air conditioning, equipment maintenance, and intelligent lighting.

First, Energy Management Optimization: Tencent utilizes AI technology to monitor and analyze the energy consumption of its data centers in real-time. By optimizing the layout of data center rooms and adjusting server workloads, Tencent has improved energy utilization efficiency and reduced energy consumption.

Second, Intelligent Air Conditioning System: Tencent has introduced an intelligent air conditioning system, using AI algorithms to precisely control the temperature and humidity of data centers. This has enhanced the efficiency of the air conditioning system and reduced energy consumption.

Third, Data Center Operations and Maintenance: Tencent employs AI technology to monitor and predict the operational status of its data center equipment. This enables timely detection and resolution of equipment failures, improving equipment reliability and stability while reducing environmental impact.

Fourth, Smart Lighting System: Tencent utilizes smart lighting systems in its data centers. Through sensors and AI algorithms, intelligent control of the lighting system is achieved, improving lighting efficiency and reducing energy consumption.

These findings provide valuable AI application scenarios for this study, aiding in a deeper exploration of AI’s role in corporate emission management. Moreover, Tencent’s environmental practices are not only innovative in technology but have also achieved significant energy-saving and emission reduction results in practical operations. By analyzing Tencent’s case, the effectiveness of AI technology in emission management is validated, offering empirical support for policymakers. Based on the above case analysis, this study designs a questionnaire survey to further explore and validate the effectiveness and potential of AI technology in enhancing corporate environmental protection practices. The questionnaire aims to collect opinions and feedback from various sources such as the public, environmental experts, and industry practitioners to evaluate the actual effects and potential value of AI applications in the environmental protection field.

The questionnaire design includes several key sections, each containing specific questions to ensure a comprehensive assessment of respondents’ understanding and attitudes toward Tencent’s environmental practices, the application of AI in environmental protection, and related management practices. Section 1: awareness of Tencent’s environmental practices: this section aims to understand whether respondents are aware of Tencent’s specific measures and achievements in environmental protection, particularly in its use of AI technology. Example questions: how familiar are you with Tencent’s use of AI technology to optimize energy efficiency management in its data centers (Very familiar/Fairly familiar/Somewhat familiar/Not very familiar/Not familiar at all)? In your opinion, what significant improvements has Tencent achieved in the application of smart air conditioning systems (Significant improvement/Moderate improvement/No change/Moderate deterioration/Significant deterioration)? Do you agree that the AI technology used by Tencent in data center operations and maintenance has improved equipment reliability and stability (Strongly agree/Agree/Neutral/Disagree/Strongly disagree)? Has Tencent’s application of AI in smart lighting systems effectively reduced energy consumption (Very effective/Effective/Neutral/Ineffective/Very ineffective)?

Section 2: evaluation of AI’s effectiveness in environmental applications: this section gathers respondents’ evaluations of the effectiveness of AI technology in environmental practices, covering improvements in various specific areas. Example questions: How would you rate the effectiveness of AI technology in optimizing energy management (Very effective/Effective/Neutral/Ineffective/Very ineffective)? Has the application of AI technology in smart air conditioning systems helped you achieve energy-saving goals (Strongly agree/Agree/Neutral/Disagree/Strongly disagree)? Do you believe AI technology has significantly improved efficiency in data center operations and maintenance (Strongly agree/Agree/Neutral/Disagree/Strongly disagree)? Has the application of AI technology in smart lighting systems significantly contributed to reducing environmental impact (Significant contribution/Moderate contribution/Neutral/No contribution/Negative contribution)?

Section 3: attitudes and recommendations for the promotion of AI technology. This section surveys respondents’ attitudes and suggestions regarding the promotion of AI technology in the field of environmental protection. Example questions: Do you support the broader adoption of AI technology for environmental protection in more companies and government departments (Strongly support/Support/Neutral/Oppose/Strongly oppose)? What measures do you think companies should take to promote the application of AI technology in environmental protection (Open-ended question, respondents can describe freely)? What policies do you think the government should implement to encourage the use of AI technology in environmental protection (Open-ended question, respondents can describe freely)?

Section 4: perception of environmental impact. This section aims to understand respondents’ views on the use of AI technology to improve the environment and achieve sustainable development goals. Example questions: how important do you think the use of AI technology is for achieving sustainable development goals in environmental protection (Very important/Important/Neutral/Not important/Very unimportant)? In your opinion, can the use of AI technology significantly improve a company’s environmental impact (Strongly agree/Agree/Neutral/Disagree/Strongly disagree)? What are your thoughts or suggestions regarding the application of AI technology in environmental protection (Open-ended question, respondents can describe freely)?

Each question uses a five-point Likert scale (Strongly agree, Agree, Neutral, Disagree, and Strongly disagree) to quantify respondents’ feedback and facilitate in-depth analysis. This approach ensures the comprehensiveness and accuracy of the data while providing rich informational support for the study.

A diverse and representative sample of respondents is selected from relevant industries and research fields, including environmental protection experts, policymakers, corporate representatives, and the general public. The selection of these respondents aims to encompass groups with a certain level of understanding and experience regarding the application of AI in environmental protection, thus providing comprehensive feedback. It is important to note that the survey does not specifically target employees of Tencent. Although Tencent is a case study with a focus on its environmental practices and AI applications, the questionnaire is not directly sent to its management team or employees. The selection of respondents emphasizes obtaining a broad perspective on the application of AI in environmental protection, rather than limiting feedback to internal opinions from Tencent. This approach ensures that the findings is more widely representative and avoids potential limitations in the sample that could affect the generalizability of the conclusions.

The questionnaire also includes questions specifically designed to assess the future development trends of AI applications in environmental protection and government support measures. Survey questions include: how do you think the application of AI technology in environmental protection will develop in the future (Significantly increase/Slightly increase/No change/Slightly decrease/Significantly decrease)? What measures do you think the government should take to support the application of AI technology in environmental protection (Increase financial support/Provide tax incentives/Formulate relevant policies/Promote corporate collaboration/Other)? Based on the survey data, this study compiles respondents’ expectations for the future development of AI technology in the field of environmental protection. It also summarizes respondents’ opinions on government support for the application of AI technology in environmental protection. For open-ended questions, qualitative analysis is conducted to summarize respondents’ specific suggestions and opinions regarding government support measures. This qualitative data provides a deeper understanding of the public’s expectations and recommendations regarding the government’s role in environmental protection.

Through expert review and pre-testing, the questionnaire content has been endorsed by professionals and understood by respondents, ensuring the comprehensiveness and accuracy of the questionnaire content. Factor analysis and correlation analysis can be used to examine the relationships between various questionnaire items and the consistency between the questionnaire items and the research framework. Meanwhile, Cronbach’s *α* coefficient is used to assess the internal consistency of each section, with values all above 0.7, ensuring high consistency of the questionnaire in various dimensions.

This study randomly selected 200 respondents for the survey by distributing questionnaires face-to-face and collected feedback from respondents. After excluding invalid responses, a total of 195 valid questionnaires were collected, achieving a response rate of 97.5%. This high response rate not only demonstrates the public’s high attention to environmental protection issues but also reflects people’s interest in and support for using AI technology to promote environmental practices. Through in-depth analysis of the questionnaire survey results, this study aims to provide empirical support for enterprises adopting AI technology in the field of environmental protection, while also providing valuable reference information for policymakers and environmental organizations.

## 3 Results and discussion

This section provides a detailed exploration of the background and methodology, particularly how the survey is employed to analyze companies’ actual operations in environmental protection and their application of AI technology. The survey results are now analyzed and discussed in detail. The following subsections focus on Tencent’s practices in environmental protection, specifically examining respondents’ understanding of these practices, which is crucial for a comprehensive evaluation of Tencent’s environmental measures and their effectiveness.

### 3.1 Understanding of Tencent’s environmental practices

**Figure 1:**
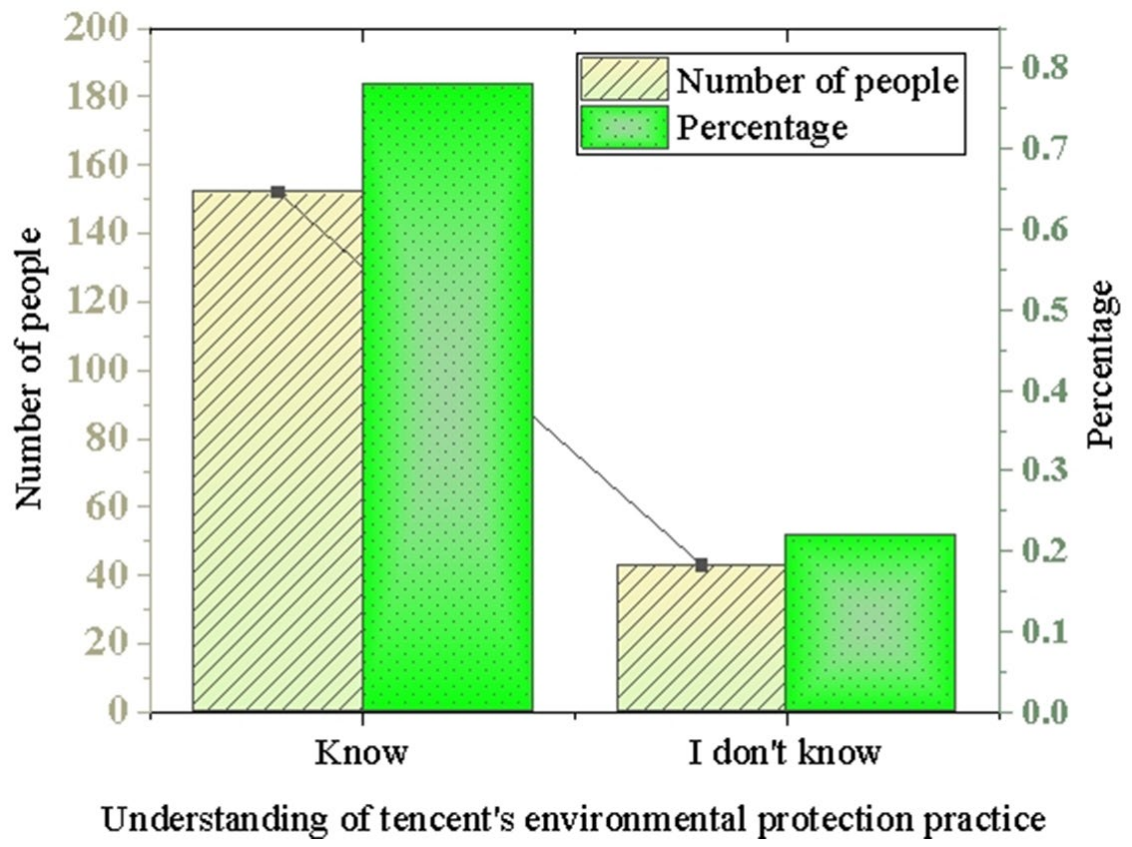
Understanding of Tencent’s environmental practices.

The experiment analyzed the understanding of Tencent’s environmental practices. The survey first revealed that the majority of respondents were aware of Tencent’s environmental practices. 78% of the respondents indicated that they were aware of Tencent’s environmental practices, while the remaining 22% stated otherwise. The conclusion suggests that Tencent’s environmental practices are to a certain extent known to the public, as shown in Figure 1.

### 3.2 Perceptions of AI application in environmental protection

**Figure 2:**
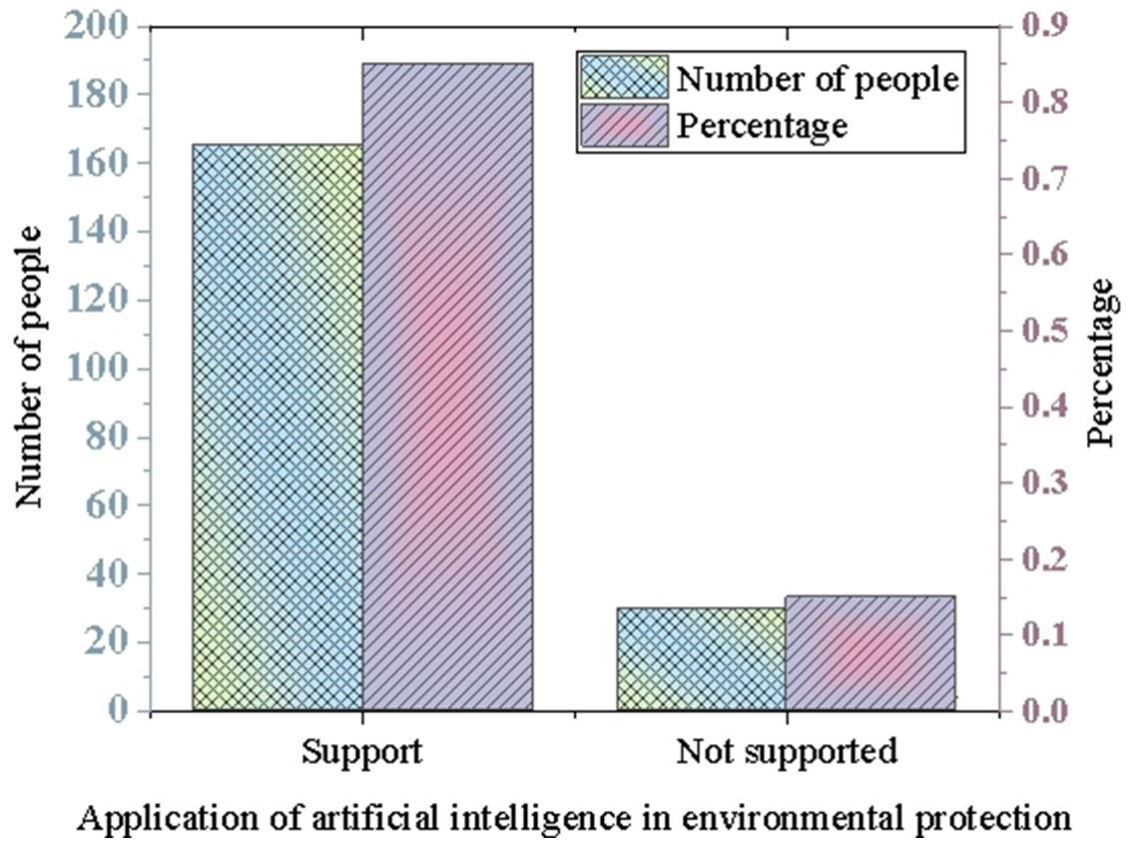
Perceptions of AI application in environmental protection.

Next, the experiment further explores respondents’ opinions and attitudes toward Tencent’s environmental practices, focusing on the application of AI in environmental protection. In the field of environmental protection, the application of AI technology is considered an important approach to addressing environmental issues. In the survey, 85% of respondents expressed support for the application of AI in environmental protection, believing that it helps improve environmental efficiency and levels. Only 15% of respondents indicated a lack of support, as shown in Figure 2.

### 3.3 Proportion of enterprises considering introducing AI technology to optimize environmental management

**Figure 3:**
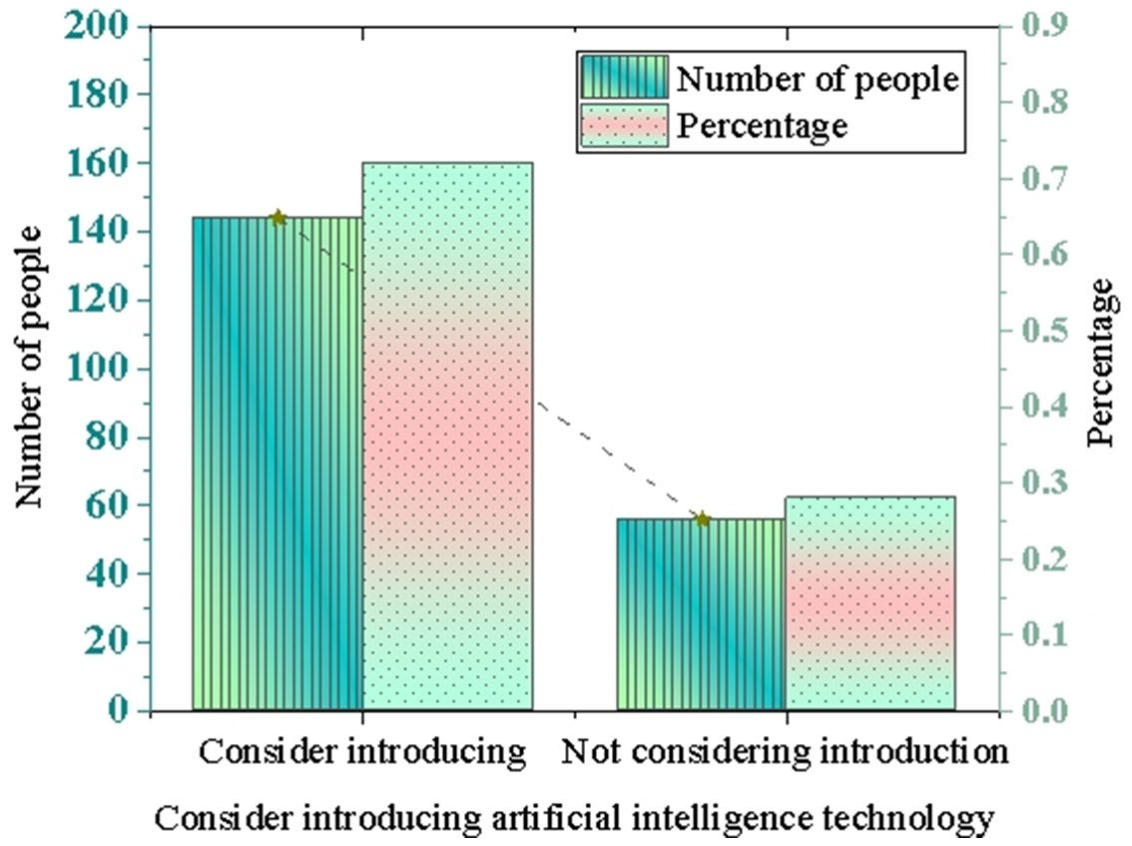
Proportion of enterprises introducing AI technology to optimize environmental management.

Whether respondents consider introducing AI technology to optimize environmental management is worthy of further exploration. In discussing the application of AI in environmental protection, the experiment also focuses on the actual actions of enterprises in this regard. The survey shows that 72% of enterprises indicate they consider introducing AI technology to optimize environmental management, while only 28% do not consider it. The data indicates that enterprises have a high level of recognition and expectations for the potential and value of AI in environmental protection, as illustrated in Figure 3.

### 3.4 Adoption status of energy management, data center operations, and intelligent lighting systems

**Figure 4:**
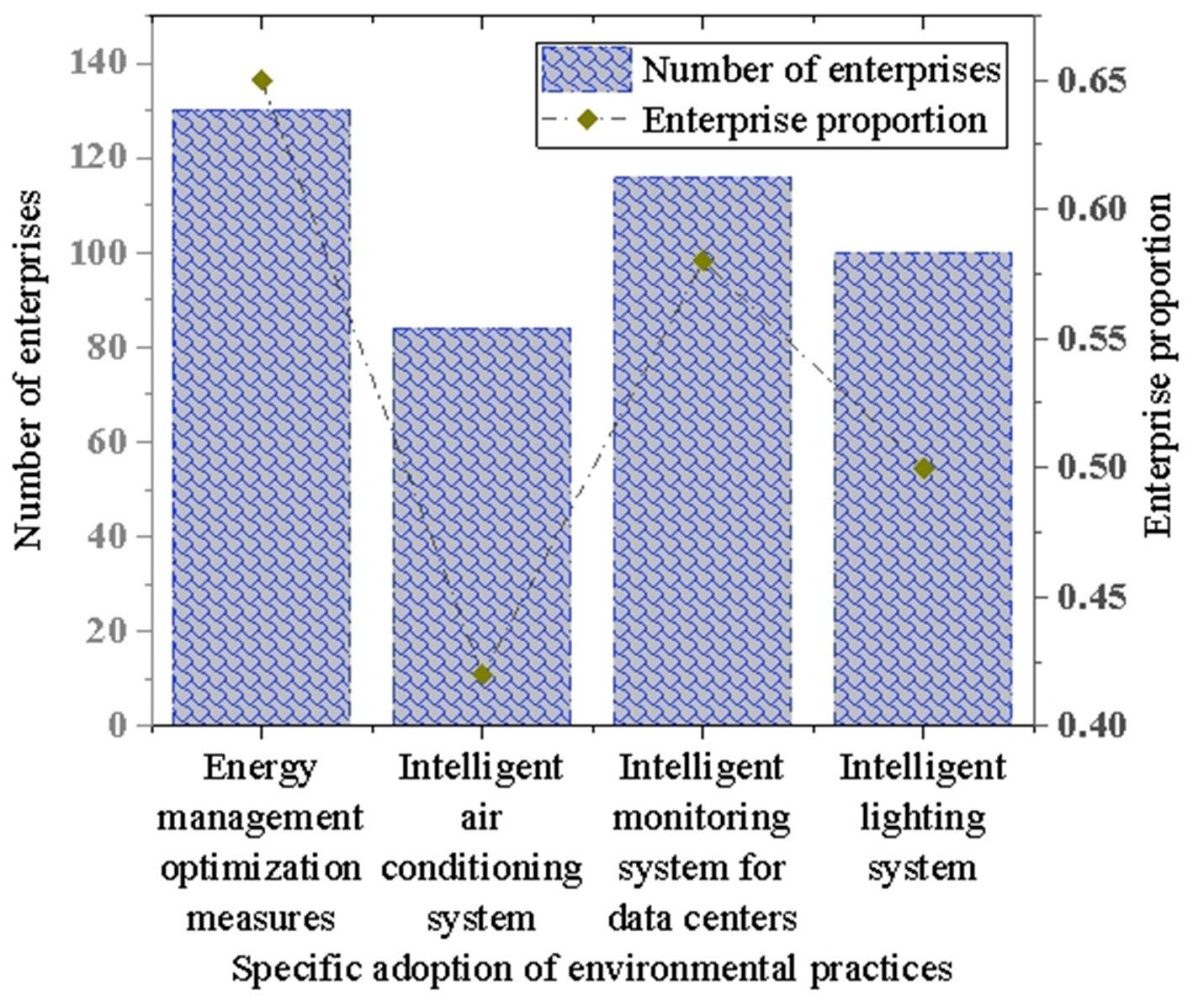
Adoption status of energy management, data center operations, and intelligent lighting systems.

The experiment further investigates the specific adoption status of enterprises in energy management, data center operations, and intelligent lighting systems. In environmental practices, the application of energy management, data center operations, and intelligent lighting systems is considered important measures to improve environmental efficiency and reduce energy consumption. The survey shows that many enterprises take corresponding measures. Specifically, 65% of enterprises adopt energy management optimization measures, 42% adopt intelligent air conditioning systems, 58% adopt intelligent monitoring systems, while 50% adopt intelligent lighting systems. These data demonstrate the proactive actions and practices of enterprises in environmental protection, as illustrated in Figure 4.

### 3.5 Views on future development and government support

**Figure 5:**
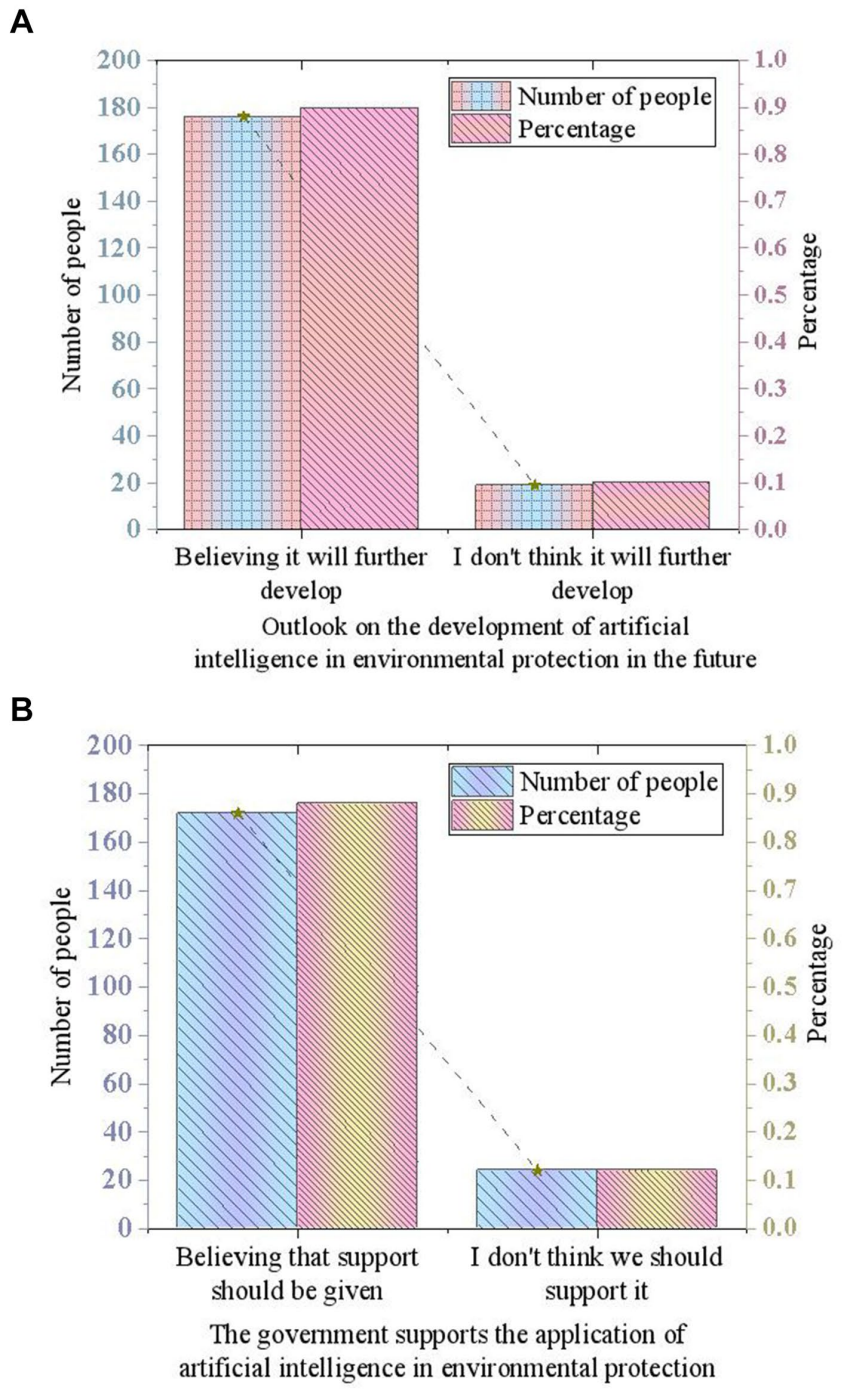
Views on future development and government support. **(A)** Views on the future development of AI in environmental protection. **(B)** Government support for the application of AI in environmental protection.

Next, the study explores respondents’ views on the future development of AI in environmental protection and government support. With the continuous development of AI technology, its application in the environmental protection field has garnered significant attention. The survey shows that the vast majority of respondents (90%) believe that the application of AI in environmental protection will further develop, indicating an optimistic outlook on the potential and prospects of AI in environmental protection. Additionally, 88% of respondents believe that the government should further support and promote the application of AI in environmental protection. These data reflect the public’s expectations for the government to play a greater role in environmental protection. Next, this study comprehensively analyzes the above survey results and discusses the prospects and development directions of AI in environmental protection, as illustrated in Figures 5A,B.

### 3.6 Impact of satisfaction assessment on Tencent’s environmental practices

**Figure 6:**
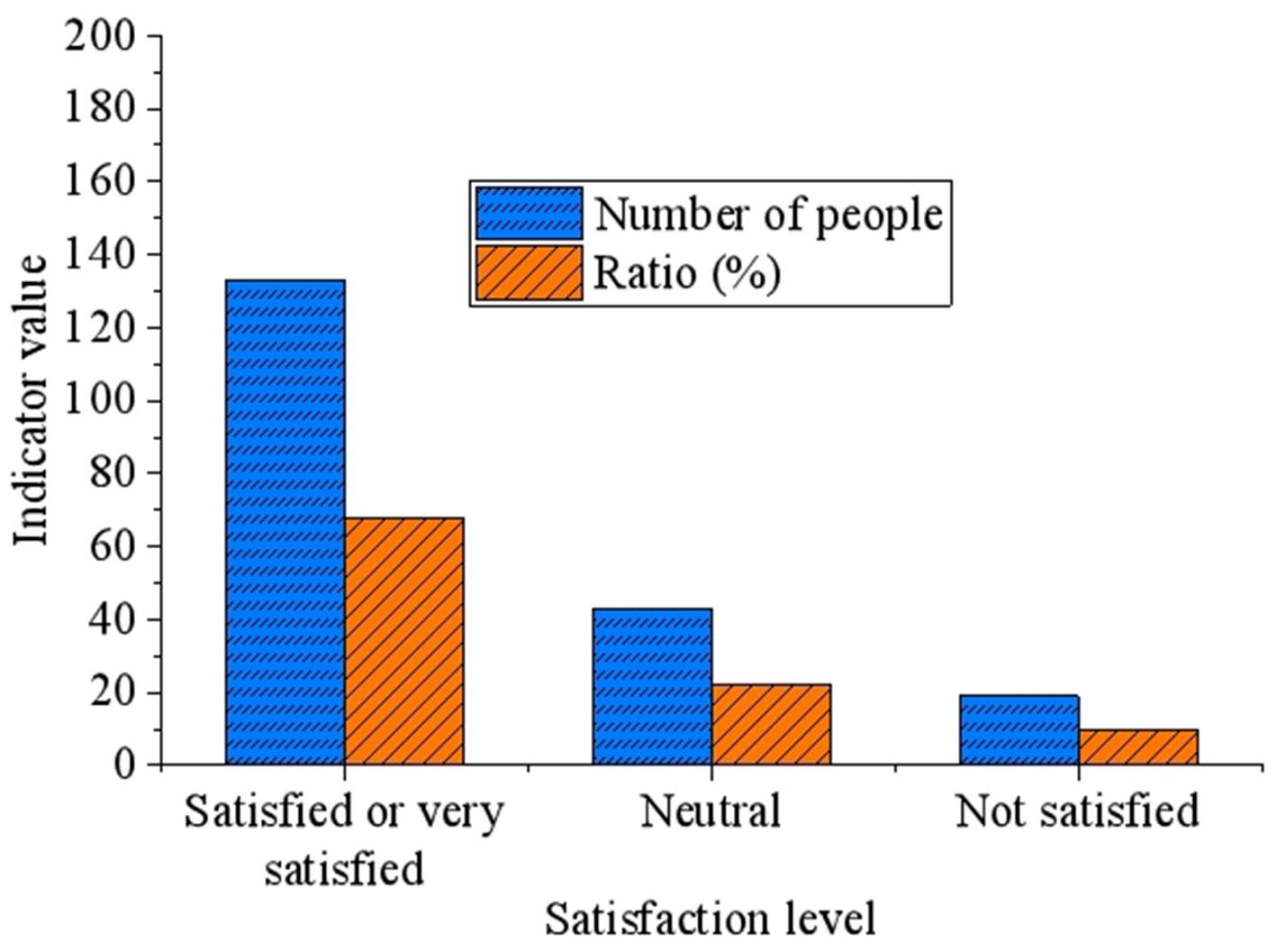
Satisfaction assessment of Tencent’s environmental practices.

To gain deeper insights into participants’ satisfaction levels regarding Tencent’s environmental practices, this study further collects data and conducts analysis (Figure 6). The survey indicates that participants generally express satisfaction with Tencent’s environmental measures. Specifically, 68% of respondents state that they are satisfied or very satisfied with Tencent’s environmental practices, 22% of respondents remain neutral, while only 10% of respondents express dissatisfaction. The data suggests that the majority of respondents recognize Tencent’s efforts and effectiveness in environmental protection.

### 3.7 Understanding the challenges and opportunities of AI application in environmental protection

**Figure 7:**
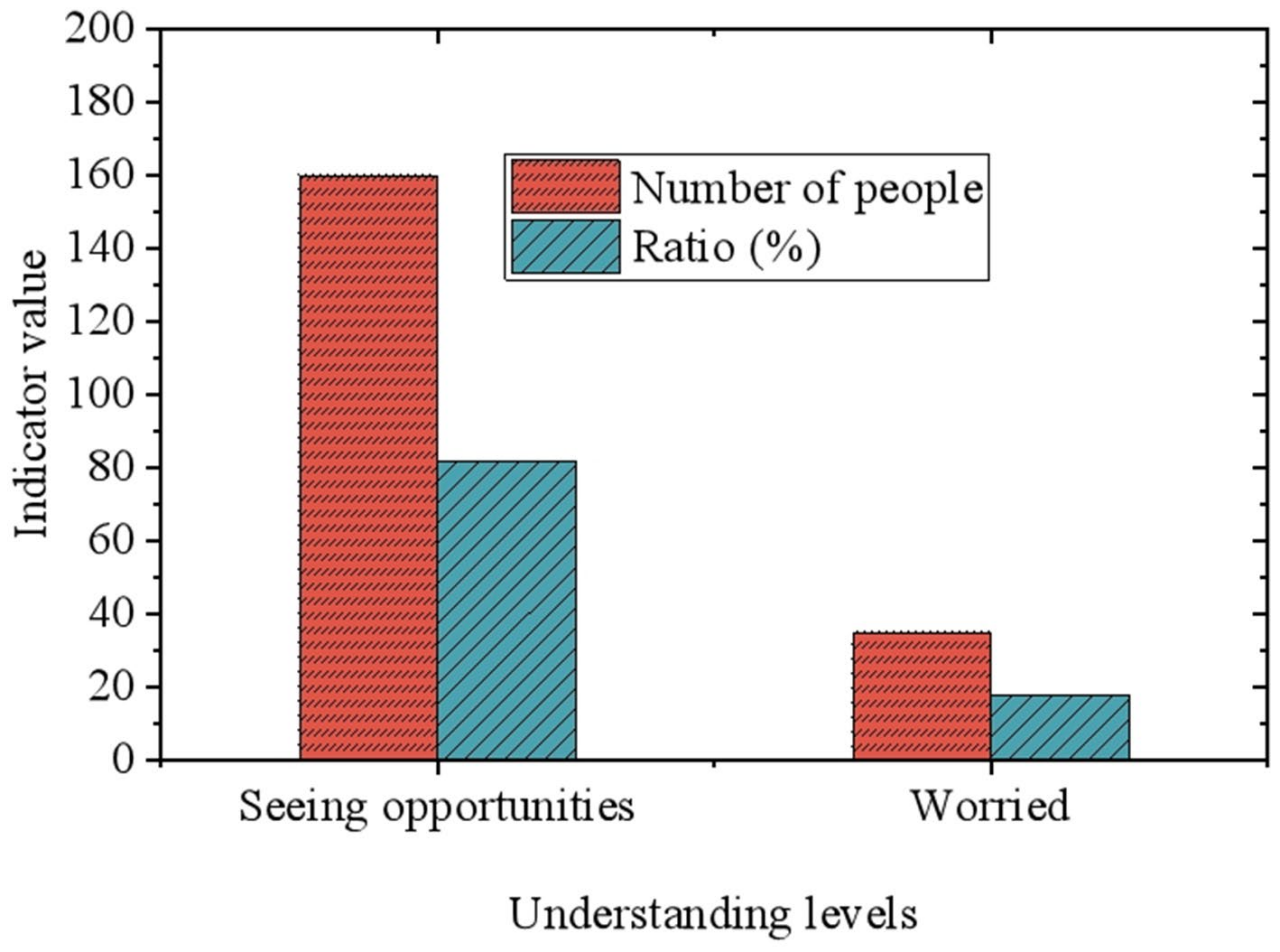
Understanding the challenges and opportunities of AI application in environmental protection.

Furthermore, the study also explores respondents’ perspectives on the challenges and opportunities of applying AI technology in environmental protection (Figure 7). The survey results indicate that while the vast majority of respondents (82%) recognize the immense potential and opportunities of AI technology in the environmental protection field, there is a certain proportion of respondents (18%) who express concerns regarding data security, technological maturity, and cost-effectiveness. These concerns highlight some of the main challenges that AI may encounter in the process of promoting its application in environmental protection.

Survey results indicate that both the public and businesses generally hold positive attitudes toward Tencent’s environmental practices and the application of AI in environmental protection. This suggests a favorable awareness and supportive atmosphere in both the technological and environmental protection domains, offering possibilities for further applications of AI technology in environmental protection in the future. However, concerns and challenges regarding the application of AI technology cannot be ignored. Issues such as data security, technological maturity, and cost-effectiveness need to be addressed during the process of promoting AI technology. Measures must be taken to address these challenges to achieve widespread application and maximum benefits of AI technology in environmental protection, including strengthening data protection, enhancing technological maturity, and optimizing cost-effectiveness ratios.

Survey results not only demonstrate widespread support from the public and businesses for Tencent’s environmental practices and the application of AI technology in environmental protection but also point out some potential challenges for future development. The results show that the vast majority of respondents hold positive attitudes toward Tencent’s environmental protection practices and express strong support for the application of AI technology in environmental protection. Data indicates that Tencent’s efforts in environmental protection and the potential of AI technology have gained recognition and expectations from the public.

However, despite the high level of support, some respondents still express concerns about the application of AI technology in environmental protection, which may be related to concerns about unknown risks associated with technological development, cost issues, and potential impacts on existing jobs. Additionally, the satisfaction assessment also indicates that there is still a certain proportion of respondents who hold neutral or dissatisfied attitudes toward Tencent’s environmental practices, suggesting that enterprises and researchers need to further explore ways to improve the satisfaction and acceptance of this segment of the population.

### 3.8 Discussion

This study investigates Tencent’s environmental practices and the application of AI in environmental protection, revealing the public and corporate perceptions and attitudes in these areas. The survey results indicate that most respondents have a high level of awareness regarding Tencent’s initiatives in environmental protection, suggesting that the company’s efforts have gained significant public attention. This reflects the effectiveness of Tencent in raising environmental awareness, but it also highlights the need for further enhancing public understanding. This aligns with Cancela-Outeda (26) discussion on the role of policy support in optimizing corporate emissions through AI, emphasizing the importance of increasing public awareness and policy backing.

The survey also shows that respondents hold a positive attitude toward the application of AI in environmental protection, believing that AI can significantly enhance environmental efficiency and effectiveness. This positive perspective is consistent with Akter et al. (27) discussion on the role of AI in climate action, indicating widespread recognition of AI technology’s potential in the environmental field. However, despite the generally positive attitude among most companies regarding the introduction of AI technology for environmental management optimization, the survey reveals that some companies have yet to take action. This highlights the practical challenges of technology implementation and emphasizes the adaptability issues under extreme climate conditions, aligning with Wang et al. (28) discussion on the role of digital technology in promoting green development.

This study specifically addresses the challenges of emissions management under diverse extreme climate conditions. The survey results show that, when faced with extreme climate conditions, companies’ environmental management strategies and the application of AI technology become particularly crucial. This is consistent with findings related to the impact of corporate green innovation, highlighting how the background of senior managers influences companies’ emission response strategies under extreme climate conditions. Extreme climate conditions impose higher demands on environmental management, necessitating more precise and flexible applications of AI technology to tackle the challenges posed by climate change.

In particular, the survey indicates that 65% of companies have implemented optimization measures in energy management, 42% use smart air conditioning systems, 58% have adopted smart monitoring systems, and 50% utilize smart lighting systems. These data demonstrate the progress companies have made in applying environmental technologies while also revealing the limitations that traditional technologies and measures may face under extreme climate conditions. The proactive actions of companies in these areas signify the potential of AI technology to enhance environmental management efficiency, but further research is needed to explore its adaptability in extreme climate scenarios.

Regarding future developments and government support, the survey results indicate that most respondents hold an optimistic view about the future of AI technology in the field of environmental protection and expect the government to provide more support. This expectation aligns with discussions about the importance of policy support, emphasizing the government’s critical role in promoting the application of AI technology. The survey also reveals a high overall satisfaction among respondents with Tencent’s environmental measures, but it highlights challenges related to data security, technological maturity, and cost-effectiveness in the application of AI technology. These challenges point to key issues that need to be addressed in the process of promoting the widespread use of AI technology.

In summary, this study demonstrates the positive attitudes of the public and businesses toward the application of AI technology in environmental protection and reveals the challenges faced during its promotion, particularly regarding its application under extreme climate conditions. These findings not only support the potential of AI in environmental management but also provide valuable insights for future research and policy formulation. To maximize the benefits of AI technology in the field of environmental protection, especially under extreme climate conditions, measures must be taken to address the current challenges, including strengthening data protection, enhancing technological maturity, optimizing cost-effectiveness, and improving the adaptability of technologies. This can promote the widespread application of AI technology, facilitating environmental protection and sustainable development.

## 4 Conclusion

This study focuses on analyzing the practical experiences of leading Chinese enterprises in the field of environmental protection, particularly regarding the application of AI technology and the role of government support. Through a systematic examination of Tencent’s environmental practices, the majority of respondents have a strong awareness of Tencent’s environmental measures and actively support the application of AI technology in environmental protection. This indicates that businesses significantly recognize the potential of AI technology in optimizing environmental management and have implemented measures such as energy management optimization and intelligent air conditioning systems to enhance environmental efficiency and reduce energy consumption. Further survey results reveal a widespread optimism about the future development of AI technology in the field of environmental protection, with respondents generally believing that it will continue to evolve. Most respondents also support the government in increasing its support for AI technology in environmental protection, viewing this as an important impetus for innovation and application in environmental protection. These findings underscore the significance of AI technology in environmental protection and present clear expectations for both the government and enterprises in promoting the application of environmental protection technologies.

Nonetheless, this study has some limitations. Firstly, some respondents have insufficient comprehensive knowledge of Tencent’s environmental practices, indicating that there is still room for improvement in public awareness. Secondly, certain enterprises have yet to adopt corresponding AI technologies, suggesting ongoing challenges in technology dissemination and application. Additionally, this study provides a preliminary exploration of the specific application effects and strategies of AI technology under different environmental conditions, and future studies need to delve deeper into these areas to comprehensively assess the actual effects of AI technology in environmental protection and the challenges it faces. To further advance research in this field, it is recommended that future work focus on exploring the application effects of AI technology under various extreme climate conditions, analyzing how policy support specifically influences enterprises’ environmental measures, and investigating the specific methods and effectiveness of enterprises in adopting AI technology. This will help provide more empirically grounded conclusions and offer more targeted recommendations for policymakers and enterprises regarding the application of environmental protection technologies.

## Data Availability

The original contributions presented in the study are included in the article/supplementary material, further inquiries can be directed to the corresponding author.
